# A Contextual Approach to Characterizing Caregiver Responsiveness in a Rural Area of The Gambia

**DOI:** 10.1111/infa.70047

**Published:** 2025-10-01

**Authors:** Helena‐Céline Stevelt, Ebrima Mbye, Ebou Touray, Tijan Fadera, Mariama Saidykhan, Ousman Kambi, Eirini Papageorgopoulou, Samantha McCann, Clare E. Elwell, Sophie E. Moore, Sarah Lloyd‐Fox, Bosiljka Milosavljevic

**Affiliations:** ^1^ Department of Psychology University of Cambridge Cambridge UK; ^2^ Medical Research Council Unit The Gambia at the London School of Hygiene and Tropical Medicine Banjul Gambia; ^3^ Department of Women and Children's Health King's College London London UK; ^4^ Department of Medical Physics and Biomedical Engineering University College London London UK; ^5^ Centre for Brain and Cognitive Development Birkbeck, University of London London UK; ^6^ Department of Biological and Experimental Psychology Centre for Brain and Behaviour Queen Mary University of London London UK

**Keywords:** majority world, maternal responsiveness, mother‐infant interactions

## Abstract

Interactions with caregivers play a crucial role in early development. While most of the world's children live in Majority World countries, research on caregiving predominantly uses measures developed in the Minority World (particularly North America and Europe), potentially biasing characterizations of parenting in understudied populations. This study describes the development of the “Demba Yaal Interaction Scale (DYIS)”, a behavioral micro‐coding scheme to assesses caregiver responsiveness in a rural, low‐resource, collectivist caregiving community in The Gambia. We adopted a contextually sensitive approach by co‐creating the scheme partnering Gambian researchers, familiar with the caregiving context, and UK researchers familiar with behavioral coding. The scheme was piloted on 5‐min videorecorded mother‐infant interactions, when infants were aged 12‐months (*N* = 50, 48% female). There were substantial individual differences in maternal responsiveness levels. Modality‐wise, responses were most likely to be non‐verbal, compared to verbal or bimodal. Mothers with some formal education were significantly more responsive and more readily engaged in bimodal responsiveness. Negative associations between these interactive behaviors and maternal demographic and socioeconomic variables (age, number of children, household size) were present but did not remain significant after correction for multiple comparisons. Moreover, associations emerged between infant physical growth and infant behaviors, as well as between maternal responsiveness and infant communication, although these too, did not remain significant after correction for multiple comparison. Our work provides a potential framework for future research seeking to develop contextually tailored assessments of caregiving practices and highlights important demographic and health variables that warrant further examination in larger samples.

## 1 Introduction

Early social interactions are foundational for infant learning, cognitive, and socio‐emotional development (Legerstee 2009). Parental responsiveness, an integral component of enriching interactions, involves parents' contingent reactions to infant cues (Beckwith and Cohen 1989; Bornstein et al. 1992, 2008). These responses serve to promote infant communication and exploration of the environment, thereby increasing learning opportunities (Prime et al. 2020; Tamis‐LeMonda and Bornstein 2002). While responsiveness is multi‐dimensional, it can broadly be considered in two forms: verbal, which involves speech, and non‐verbal behavioral, which involves a range of physical actions that occur in response to infant activity (Alvarenga et al. 2021; Masur et al. 2005; Tamis‐LeMonda et al. 2001; Tamis‐LeMonda and Bornstein 2002). Moreover, responsiveness is dynamic across infant development, with caregivers engaging in different behaviors to match infants' increasingly sophisticated social and communicative abilities (Bornstein et al. 2008).

Among infants who are exposed to environmental adversity, heightened parental responsiveness has been associated with better cognitive and behavioral outcomes (for review see Korom and Dozier 2021). These findings have gained attention in global health research, as statistics suggest that over 250 million children worldwide are exposed to poverty‐related factors that undermine their cognitive development (Grantham‐McGregor et al. 2007). Consequently, parenting interventions to increase caregiver responsiveness are increasingly being implemented, particularly in low‐ and middle‐income countries (LMICs), where poverty related adversity is more prevalent (Pedersen et al. 2019). While showing promise, such interventions are largely based on evidence of developmental mechanisms and parenting practices from “Minority World” contexts, such as Europe and North America (Kidd and Garcia 2022; Nielsen et al. 2017; Singh et al. 2023; Tomlinson et al. 2014). This raises important concerns that promoting foreign practices may negatively affect culturally organized patterns of childcare in local communities, ignoring the possibility of alternative pathways in development (Casillas et al. 2020; Morelli et al. 2018; Ochs and Kremer‐Sadl 2020; Serpell and Nsamenang 2014). Furthermore, most of the world's children are growing up in “Majority World” contexts (which includes countries in the Global South and those categorized as LMICs) and characterizing parenting practices through the lens of a subset of the world's population is not necessarily representative of their experiences (Draper et al. 2023). A recent review by Bozicevic et al. (2024) highlights that, while coding schemes of maternal responsiveness developed in the Minority World have been implemented in Majority World settings, there is a scarcity of measures developed specifically for use in these contexts. This limits our capacity to understand the relationship between, and mechanisms underlying, caregiving and child outcomes within individual cultures in the Majority World.

Contingent responsiveness (i.e., prompt and appropriate caregiver responses to infant cues) is a common characteristic of parenting worldwide, with mothers in different countries displaying similar levels of responsiveness (Bornstein et al. 2008; Broesch et al. 2016; Tamis‐LeMonda et al. 2014). However, parenting behaviors may be organized and expressed differently in different contexts (Bornstein et al. 1992; Bradley and Corwyn 2005; Cristia 2022). The form, content and timing of parent‐infant interactions are shaped by assumptions about infants' ability to communicate, parents' socialization goals, norms about infants' role in social interactions and how adults should interact with children (Bornstein and Landsford 2012; Keller et al. 2005; Kuchirko and Tamis‐LeMonda 2019). The predominant modalities in parent‐infant interactions in Minority World contexts are mutual gaze and vocalization (Gratier and Devouche 2017; Northrup and Iverson 2020). On the other hand, there is evidence that parent‐infant interactions in Majority World settings involve more physical contact over visual cues and place less emphasis on face‐to‐face didactic conversations (Gratier and Devouche 2017; Kuchirko and Tamis‐LeMonda 2019; Little et al. 2016; Serpell 2017).

Differences in the constituent responsiveness behaviors do not imply disparities in their ability to promote healthy infant outcomes (Tamis‐LeMonda et al. 2014). On the contrary, context‐specific parental behaviors demonstrate the rules of social interactions and appropriate behaviors for a particular context. Therefore, regardless of parents' specific behaviors, responding to infant cues creates opportunities for learning (Zhang et al. 2021). However, if emphasis is placed on parenting behaviors derived solely from Minority World settings, this may result in misclassifying parents from other contexts as less responsive or missing behaviors that are crucial for infant development. A substantial proportion of the research on parenting is focused on verbal responsiveness (Rocha et al. 2020), which may not be appropriate in Majority World contexts, particularly in rural communities, where non‐verbal behaviors play an important part in parent‐infant interactions. For instance, 3–10 month‐old infants in rural societies in Kenya and Cameroon experience more proximal parenting, involving physical contact and stimulation, whereas infants in Greece and the USA experience more distal parenting, emphasizing face‐to‐face exchanges, infant‐directed talk, and object stimulation (Keller et al. 2004; Richman et al. 1992).

These differing parenting styles arguably promote skills that are relevant for the infants' context (Bornstein and Landsford 2012). For example, foundational work by Levine (1989) that compared caregiving practices between Kenyan Gusii and American mothers demonstrated that Gusii mothers' highly rapid responses to infant distress meant that infants in this community cried less at 3–4‐month, while American mothers' propensity to use verbal responses was proposed to promote more communicative behaviors among American infants. Similarly, 18–20‐month‐old Nso infants in Cameroon were found to develop self‐regulation earlier when compared to infants in Greece due to the enhanced physical contact characteristic of their caregiving environment at 3‐months old, while the Greek infants developed self‐recognition at 18–20 months, earlier than the Nso infants, due to greater exposure to speech and conversation at 3‐month of age (Keller et al. 2004). Findings related to the importance of verbal responsiveness appear to be context specific. For example, in a low‐income sample in Brazil, 11–18‐month‐old infants whose mothers engaged in more bimodal (combining verbal and non‐verbal) responsiveness exhibited more communicative behaviors (Alvarenga et al. 2021). However, among the Tsletal Maya in Mexico, where infant‐directed speech is rare, 5.5–11‐month‐old infants are capable of learning language largely through overhearing, thus challenging the notion that infant directed speech is the sole mechanism for language learning (Foushee and Srinivasan 2024).

Taken together, this evidence suggests that it may not be appropriate to evaluate caregiver responsiveness in a particular culture by simply transplanting measures developed in contrasting contexts. Moreover, while we use the dichotomy of Minority and Majority World countries to highlight this disparity, it is important to recognize the vast heterogeneity of cultures, norms, and parenting practices in both contexts. Even within cultures, individual and demographic characteristics, such as socioeconomic status (SES), caregiver education (Richman et al. 1992), number of children caregivers have (McFadden and Tamis–LeMonda 2013McFadden and Tamis‐LeMonda 2013; Obradović et al. 2017) and infant behavior and health (Harel‐Gadassi et al. 2020; Takács et al. 2020), impact on dyadic interactions. Thus, rather than employing measures developed for different Majority World contexts, it is crucial to construct more context‐specific measures of caregiver responsiveness, that incorporate local norms, to facilitate the development of parenting support tools for vulnerable children around the world.

### 1.1 Study Context

The current study was done as part of the Brain Imaging for Global Health (BRIGHT) project (www.globalfnirs.org/the‐bright‐project) a prospective longitudinal study of neurocognitive development over the first 5 years of life in the West Kiang region of The Gambia (Lloyd‐Fox et al. 2024). West Kiang is a rural community where a majority of the population are subsistence farmers (Hennig et al. 2017). Qualitative work suggests that the population hold strong religious, community, and family values (Brotherton et al. 2021; Sear and Mace 2009; Sosseh et al. 2023). Families tend to live in multi‐generational households, with extended kin, where childcare is viewed as a shared responsibility (Brotherton et al. 2021; Kea 2013; Sear and Mace 2009). Multiple contextual adversities pose challenges for the community in West Kiang and compromise child wellbeing (Hennig et al. 2017; Van Der Merwe et al. 2013). Most notably, the annual rainy and dry seasons result in fluctuation in farming productivity and, thus, the availability of diverse and nutritious foods throughout the year (Hennig et al. 2017). Most of the population live below the poverty line and educational opportunities and literacy are low among adults, especially women (Hennig et al. 2017). These factors, alongside the high prevalence of infectious disease (Hennig et al. 2017), have been shown to contribute to growth faltering among infants during the first year of life (Schoenbuchner et al. 2019). Undernutrition and poorer physical growth have, in turn, been identified as contributing to poorer infant neurocognitive development (Bulgarelli et al. 2025; McCann et al. 2023; Milosavljevic et al. 2019).

However, the Gambian government has been making strides to ensuring access to primary and, more recently, preschool education for children in the country (Blimpo et al. 2022; The Gambia Bureau of Statistics 2011). In line with this, psychoeducational programs, aimed at teaching parents about the importance of social stimulation, have been piloted and have demonstrated some effectiveness (Blimpo et al. 2024). This highlights the ways in which local initiatives to promote cognitive development through parenting interventions are becoming a priority, led by national organizations. To support such initiatives, it is crucial to establish contextually appropriate measures of parenting characteristics and outcome measures to evaluate their effectiveness.

### 1.2 Aims of the Current Study

The battery of assessments in the BRIGHT project involved collecting data on maternal‐infant interactions during recorded, free play sessions administered at, 1‐, 5‐, 8‐, 12‐, 18‐, and 24‐months of age (Lloyd‐Fox et al. 2024). In the present study, we use data collected at the 12‐month time point to develop a contextually sensitive behavioral coding scheme of caregiver responsiveness for this setting. We named the scheme the “Demba Yaal Interaction Scale (DYIS)”, using the Mandinka word for family (demba yaal).

The onset of the second year of life is important as it marks substantial growth in motor, cognitive, and language milestones and, consequently, it is also the age when neurodevelopmental vulnerabilities start to become outwardly observable (Brian et al. 2014; Karasik et al. 2014; Yaari et al. 2018). However, prior work examining caregiver‐infant interactions in global contexts has largely focused on very early infancy (e.g., Bozicevic et al. 2021). Therefore, we sought to fill this gap in the literature and capture caregiving practices at this developmentally pivotal point in infancy. The aim of the present study is to describe the development of this behavioral coding scheme of caregiver responsiveness. Furthermore, we use a subset of the BRIGHT participants to pilot this coding scheme to characterize maternal and infant behaviors and to examine whether contextually specific demographic and SES (lower educational attainment, larger number of children, larger household size) and poorer infant health (reduced physical growth) factors negatively impact on mother‐infant dyadic behaviors.

## 2 Methods

### 2.1 Participants

Families were recruited during an antenatal clinic visit at the Medical Research Council Unit The Gambia at the London School of Hygiene and Tropical Medicine (MRCG at LSHTM; https://www.lshtm.ac.uk/research/units/mrc‐gambia) field station in Keneba, West Kiang. To avoid confounds with linguistic translation of assessments, only families of the Mandinka ethnic group, the majority in the region (Hennig et al. 2017) were recruited. The BRIGHT sample consisted of 222 families; infants were included if they were born at 37–42 weeks' gestation and not diagnosed with any neurological difficulties postnatally. For full detail of BRIGHT recruitment and study visits please see Lloyd‐Fox et al. (2024). To pilot the coding scheme, a subset of 50 mother‐infant dyads (48% female) was randomly selected from the full sample that attended the 12‐month visit (*N* = 188). This sample size was based on published studies using similar coding of maternal responsiveness, which ranged from 20 to 40 participants (Bornstein et al. 1999; Kärtner et al. 2008; Masur et al. 2005; Richman et al. 1992; Tamis‐LeMonda et al. 2001). The BRIGHT project was approved by the joint Gambia Government/MRC Unit The Gambia Ethics Committee (SCC 1351) and full informed consent was obtained from all participating families prior to data collection.

### 2.2 Maternal‐Infant Interaction Study Design

**FIGURE 1:**
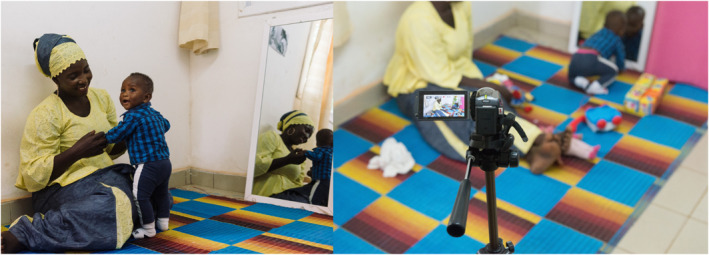
Set up of the maternal‐infant interaction recordings in the no‐toy (left) and toy (right) sections. Photo credit: Ian Farrell.

Mother‐infant dyads took part in a 10‐min videotaped, free‐play session. They were seated on a mat in the corner of a white‐walled room, and videos were recorded with two cameras positioned at a 90° angle to each other. A large mirror was propped against one wall, to facilitate viewing of participants during recording when they were turned away from the cameras (see Figure 1). Mothers were asked to interact as they normally would at home. The first 5 min involved free play without toys (no‐toy section), after which an assortment of toys (the same for each participant) was introduced (toy section).

### 2.3 Development of the Demba Yaal Interaction Scale (DYIS)

We sought to develop a coding scheme that would quantify levels of caregiver responsiveness by evaluating specific sequences of caregiver‐infant interactive behaviors (i.e., what infants did and if, and how, caregivers reacted). The coding scheme was co‐created between the BRIGHT project researchers in the UK (who were familiar with existing behavioral coding schemes for this age range) and researchers in The Gambia (who are highly experienced in caregiving in this community, infant neurocognitive assessments and are native Mandinka speakers). Consultation meetings followed an iterative process that involved meeting to discuss the conceptual framework, selection of contextually appropriate caregiver/infant behaviors, and joint viewing of videos to ensure consistency in coding. The emphasis was to incorporate insight of local perspectives on parenting, and to capture the full range of behaviors that mothers and infants might display.

#### 2.3.1 Methodological Framework

One methodological decision was whether to implement a micro‐level (coding caregiver behaviors that occur in small time segments) or macro‐level (assigning behaviors a global score based on the full duration of the interaction) coding system (Mesman 2010). The micro‐level approach was selected because it generates a specific count/frequency of behaviors, is less reliant on subjective coder judgment, minimizes cross‐cultural bias, and allows for investigation of specificity of behaviors (Chorney et al. 2015). While levels of maternal responsiveness may be similar across cultural settings, the types of caregiver responses and the infant cues responded to differ (Bornstein et al. 1992; Broesch et al. 2016), and a micro‐coding scheme enables capturing this specificity. Furthermore, a dichotomous coding procedure (i.e., only marking when behaviors occurred) was used instead of continuous/ordinal rating scales (Chorney et al. 2015).

An additional consideration was whether to code the toy or no‐toy section. Initial viewing of the videos uncovered that dyadic interactions became minimal upon the introduction of toys. Prior research suggests that manufactured toys are not readily available in rural, low‐resource Majority World settings (Bradley and Corwyn 2005), which the Gambian team confirmed was also the case in West Kiang. Therefore, we concluded that using manufactured toys was not representative of the participants' home environment or daily interactions and focused the coding on the no‐toy section.

#### 2.3.2 Identification of Caregiver and Infant Behaviors for Coding

The development of the coding scheme drew inspiration from other micro‐coding schemes used in rural, low‐income, Majority World settings (e.g., Alvarenga et al. 2021; Bornstein et al. 1992; Broesch et al. 2016; Keller et al. 2004; Richman et al. 1992), rather than transpose a full scheme developed for a different cultural context and age group. Initially, a list of target behaviors was generated by adapting a sweeping list of caregiver and infant behaviors by Clarke‐Stewart (1973). This list was chosen for two reasons: firstly, it supplied a starting point of naturally occurring behaviors (23 infant and 26 maternal behaviors) from which we could adapt our coding scheme and incorporate additional behaviors or modifications recommended by the Gambian researchers. Secondly, this list had already been adapted for cross‐cultural micro‐coding research by Richman et al. (1992) when coding mother‐child interactions among the Gusii of Kenya, demonstrating its potential as a starting point for developing a micro‐coding scheme in a cross‐cultural setting. The adaptation involved (i) excluding behaviors not applicable to the current recording set‐up (e.g., putting the infant in a high‐chair), (ii) minor wording changes to operational definitions, and (iii) combining speech‐content codes to fit the young age of the participants and to reduce reliance on interpreting adult speech. A full list of infant and caregiver behavior codes and operational definitions are presented in Table 1.

**TABLE 1:** Overview of caregiver and infant target behaviors used in the coding scheme.

Behavior	Operational definition
Infant behaviors	
Exploratory	
Look	Looks at person or object (min. 2 s)—looks only, not during play with target
Look to caregiver	Looks at caregiver's face (regardless of duration), when not doing anything else with the caregiver.
Play	Manipulates, sucks, bounces, bangs, examines, etc., mother, clothes, or an object.
Communicative	
Vocalize	Makes a vocalization—speech or pre‐speech—or imitates mother’s vocalization
Fuss/cry	Whines, cries, or makes other negative vocalizations
Smile	Smiles or laughs
Gesture	Reaches or points towards object or person
Caregiver behaviors	
Non‐verbal behavioral responses (non‐speech)	
Touch—affectionate	Touches hand to infant, or more deliberate holding of infant (puts arm around or hugs infant, physically soothes infant) or other tactile affectionate action (kisses, caresses, fondles, ruffles hair of, cuddles infant)
Touch—play	Engages in deliberate, active, physical stimulation (e.g. rocks, bounces, tickles, plays physical game with infant) or touches infant to provide physical support.
Reposition infant	Repositions, picks up, or puts down infant
Play	Entertains infant with fingers or hands (e.g. finger‐snap, clap), or object/clothing, plays game.
Look	Moves gaze to look at infant—when not doing anything else with them
Smile	Smiles at infant (nonverbal expression)
Confirmatory act	Nods, waves, or makes other distal, non‐verbal acknowledgement of infant's behavior
Point	Points to an object or person.
Social sounds (non‐speech)	Whistles, whispers, laughs, verbally soothes, clicks tongue, hums (“hmm”)
Verbal responses (speech)	
Vocalization	Speech or song directed at infant or imitates infant's vocalization.

Infant behavior codes were then divided into exploratory and communicative behavior categories. *Exploratory* behavior codes included “Look”, “Look to caregiver” and “Play”, while *communicative* behavior codes included “Vocalization”, “Fuss/cry”, “Smile/laugh”, and “Gestures”. In line with this, the Gambian researchers identified facial expressions (such as smiling), fussing, and movement towards objects/people as infant behaviors that parents consider communicative. The decision to include “Fuss/cry” as a communicative behavior was based on prior work (e.g., Alvarenga et al. 2021; Mesman 2010; Bornstein et al. 1992), suggesting that infant distress signals are an important communicative tool that may serve to elicit intuitive parenting behaviors. On the other hand, moving towards objects/people was excluded later during piloting as maternal responses to this behavior were disrupted by the video recording (i.e., mothers physically moved/repositioned infants back in view of the cameras). Infant “gesture” was added to the list of communicative behaviors by merging codes for infants *showing* objects and *pointing/reaching towards* objects, and changing the operational definition to include infants reaching out towards objects *without extending their index finger* (Cameron‐Faulkner et al. 2015, 2021).

Caregiver behaviors were not limited to those used in existing micro‐coding schemes, as prior work was conducted in different cultural/physical contexts (e.g. Japan, Bornstein et al. 1992; Brazil, Alvarenga et al. 2021; Kenya, Keller et al. 2004; Kenya and Fiji, Broesch et al. 2016) and/or relied strongly on verbal responses (Bornstein et al. 1992, 2008). Instead, a wide range of caregiver behaviors was maintained from the list in Clarke‐Stewart (1973). Behaviors that were penalizing (e.g., physically disciplining, restraining, or scolding the infant) or those not referring to objectively observable behaviors (e.g., coding whether a response was “appropriate”), were excluded. The final caregiver response behavior codes for *non‐verbal behavioral responses* included “Touch (affectionate)”, “Touch (play)”, “Reposition infant”, “Play”, “Look to infant”, “Smile”, “Confirmatory act”, “Point”, “Social sounds (non‐speech)”, and for *verbal responses* “Vocalization”, including any speech or song (see Table 1). Modifications included combining the different caregiver touch codes from Clarke‐Stewart (1973) into two codes: “touch (play)” and “touch (affectionate)”. Moreover, the Gambian researchers identified finger‐snapping as a behavior adults use to entertain infants, and this was added to the “play” code. The operational definition for “look to infant” was redefined to include turning of gaze to infant (rather than continued looking), as a response must involve *change* in behavior (Bornstein et al. 1992, 1999). Upon recommendation from the Gambian researchers, confirmatory behaviors such as nodding or “thumbs up” were added as a category—“Confirmatory act”. Additionally, the code for showing was renamed to “Point” to more accurately reflect the physical pointing behavior specified in the definition. An additional recommendation by the Gambian researchers was to add humming (“hmmm”) and tongue‐clicking to the operational definitions for the “social sounds (non‐speech)” code. Furthermore, they described how parents speak to and modify their language around infants, clarifying the nature of verbal responsiveness in this population.

### 2.4 Coding Procedure

Coding was done by a single experimenter from the UK team (HCS) using Datavyu (v1.3.7, Datavyu Team 2014, RRID:SCR_003587). Subsequently, 20% of videos (*N* = 10) were coded by a second trained coder (EP) based in the UK, who was not part of the original coding scheme development. Inter‐rater reliability was assessed using intraclass‐correlation coefficient (ICC), based on a single measurement, two‐way random effects model with consistency, ICC (C, 1). ICC (C, 1) estimates for number of infant exploratory, communicative, and overall number of behaviors ranged from 0.70 to 0.94, indicating moderate to excellent reliability, while ICC (C, 1) estimates for maternal responsiveness derived from the behavioral coding (see below) ranged from 0.77 to 0.97, indicating good to excellent reliability (Koo and Li 2016; Liljequist et al. 2019).

Coding was done in two passes; first, a continuous coding pass was done to identify all relevant infant behaviors by marking their onset. Then, 5‐s windows were defined from the onset of each infant behavior. This was followed by a time‐segment coding pass to identify maternal response behaviors, where the presence or absence of a response to the infant's behavior, and the specific behavior the mother used to make a response, were recorded within each 5‐s window. The 5‐s duration was chosen based on prior studies that defined a response within this time frame as being contingent (Bornstein et al. 1992, 1999, 2008; Tamis‐LeMonda et al. 1998; Tamis‐LeMonda et al. 2001). Consequently, for infants, each behavior could occur spontaneously or in response to maternal behaviors, while maternal behaviors were only recorded if they occurred within 5‐s of the onset of an infant behavior. If further infant behaviors occurred within the same 5‐s response window, maternal behaviors were coded as the response to the most recent infant behavior. This ensured maternal responses were counted only once. If multiple infant behaviors occurred within 1‐s of each other (e.g., vocalizing and playing), they were considered as a single infant behavioral event for subsequent analyses.

The codable video length differed between mother‐infant dyads, with some lasting shorter or longer than 5‐min. Based on prior literature, videos were deemed too short if the duration was under 3‐min (Müller et al. 2019). The average codable video length was 4 m 51 s (SD = 21 s), with only one that was shorter than 4 min at 3 m 05 s. For some infant behavioral events, the mother was not visible within the videoframe during the 5‐s response window. These events were used in examination of infant activities but excluded from analyses of maternal responsiveness.

To account for varying video lengths, raw infant behavior frequencies were converted into counts per minute. Raw counts of maternal responses differed between mothers because of both video length and infant activity levels (i.e., mothers with less active infants had fewer opportunities to respond). Thus, raw counts of maternal responses were converted to *proportions* relative to the number of behavioral events their infants displayed. The constituent behaviors of the maternal responses were further classified by modality as *verbal* (containing only speech), *non‐verbal* (any non‐speech behavior[s]), or *bimodal* (integration of verbal and non‐verbal behaviors) and represented as *proportions* of the total number of responses.

### 2.5 Maternal Demographic Characteristics

Maternal demographic variables were collected using questionnaires at the 7–14‐day visit. Factors included in the current study were mothers’ age, number of children, household size (by asking how many adults and children lived in their household), and formal education. Maternal education was examined both as number of years in school and, because many mothers did not have any formal schooling, a dichotomous variable indicating whether the mother had any formal education (yes/no).

### 2.6 Infant Anthropometric Measures

Infant weight and length were collected at the 12‐month visit. Length was measured with a Harpenden Infantometer length board (Holtain Ltd.) with a precision of 0.1 cm and weight was measured using an electronic baby scale (Model 336, SECA) with a precision of 0.01 kg. Measures were taken three times and the mean of those were used in analyses. Infants' length‐for‐age and weight‐for‐length sex‐adjusted *z*‐scores were computed according to WHO reference norms (WHO Multicentre Growth Reference Study Group 2006). These scores can be used to identify infants that are stunted (length‐for‐age *z*‐score < −2 SD) or wasted (weight‐for‐length *z*‐score < −2 SD).

### 2.7 Data Analysis Strategy

The first objective in data analysis was to comprehensively characterize maternal and infant behaviors. For infants, we examined overall activity levels, rates of communicative and exploratory behaviors, and rates of each constituent behavior. For maternal behaviors, we examined overall responsiveness, rates of verbal, non‐verbal, and bimodal responsiveness, and all constituent behaviors. Friedman's test was used to compare the rates of responses in each modality. As a significant difference emerged (see results) pairwise Wilcoxon signed rank tests were used to identify the modalities that significantly differed from each other.

To examine the contribution of physical growth to infant behavior, correlations were run between infant weight‐for‐length and length‐for‐age *z*‐scores, the number of exploratory and communicative behaviors. As these analyses are exploratory, we also ran correlations with each constituent behavior. To control for potential effects of infant distress on the relationship between physical health and communication, a supplementary analysis was run excluding fussing from the communicative behavior category. All variables were checked for normality, and if both variables fit assumptions of normality, Pearson's correlation was run, otherwise non‐parametric Spearman's rho correlations were used.

To assess the impact of maternal education on responsiveness, Mann–Whitney U tests were run to compare overall responsiveness and each response modality between mothers who had some versus no formal education. For mothers who had some formal education, Spearman Rho correlation was used to examine the association between the number of years of schooling and overall responsiveness. Similarly, Spearman Rho correlation was used to examine the association between maternal age, number of children, household size and overall maternal responsiveness. Finally, Spearman Rho correlations were used to assess associations between overall maternal responsiveness, response modalities and infant number of exploratory and communicative behaviors. Given the exploratory nature of these analyses, significance is reported using both uncorrected and Bonferonni‐adjusted *p* values.

### 2.8 Transparency and Completeness of Methods

We used the Strengthening of the Reporting of Observational Studies in Epidemiology checklist (STROBE; von Elm et al. 2007) to assess the transparency and rigor of our approach in developing this scale and the way in which our findings are reported. The full checklist can be found in Supporting Information S1.

## 3 Results

### 3.1 Demographic Characteristics

Maternal demographic characteristics and infant anthropometric scores are summarized in Table 2. There was an equal number of male (*N* = 26) and female (*N* = 24) infants (*X* ^2^ (50) = 0.08, *p* = 0.777). Mean length‐for‐age *z*‐score was −1.04 and mean weight‐for‐length *z*‐score −0.55; 6 infants were stunted and 4 wasted (no overlap between stunted/wasted).

**TABLE 2:** Maternal and infant demographic characteristics and infant anthropometric scores.

	*N*	Mean (SD)	Range
Infant			
Age at visit (months)	50	12.19 (0.46)	11.60 to 14.06
Length‐for‐age *z*‐score	50	−1.04 (0.89)	−3.80 to 0.78
Weight‐for‐length *z*‐score	50	−0.55 (1.00)	−2.58 to 1.87
Mother			
Age at childbirth (years)	50	30.73 (6.93)	20.01 to 42.60
Number of children	41	5.02 (2.56)	1 to 9
Household size	41	14.00 (7.29)	5 to 36
Formal education	41	—	—
No formal education	25	—	—
Any formal education	16	—	—
*If any formal education,* number of years		7.5 (3.67)	1 to 12

*Note:* Household size was measured by asking mothers how many individuals were lived in the family household.

### 3.2 Characteristics of Maternal‐Infant Interactions

**FIGURE 2:**
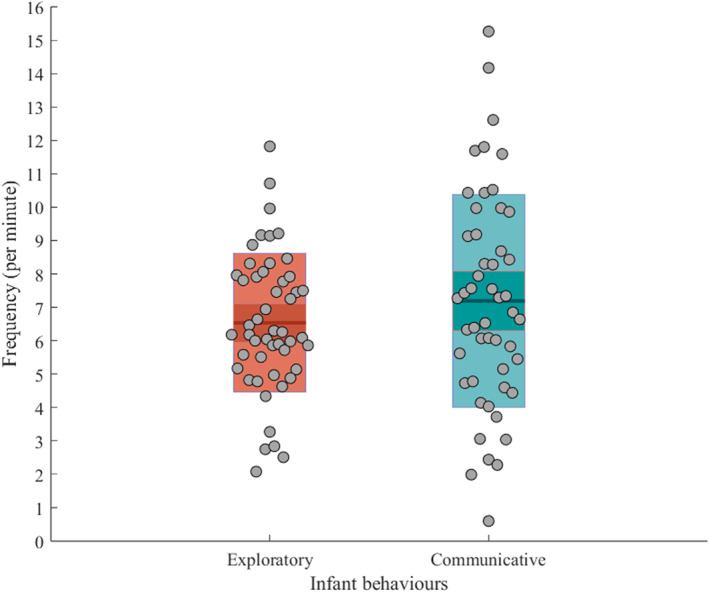
Frequency of infant exploratory and communicative behaviors. The middle lines represent the group mean, the inner bands represent the 1.96 standard error of measurement intervals, and the outer bands the ± 1 standard deviation intervals. The gray dots represent individual data points.

Table 3 and Figure 2 present an overview of infant behaviors. These behaviors were distributed across *M* = 11.84 (SD = 2.57) behavioral events per minute, as there were instances where two or more infant behaviors occurred simultaneously.

**TABLE 3:** Frequency of infant behaviors (per minute)—all coded behaviors, occurrences of multiple behaviors simultaneously, exploratory and communicative behaviors, and constituent behaviors, including Shapiro–Wilk test statistics, *N* = 50.

	Mean (SD)	*W* (*p* value)	Min	Max
	freq./min			
All coded behaviors	13.72 (3.64)	—	7.11	21.24
Occurrences of multiple behaviors simultaneously	1.85 (1.29)	—	0.00	5.06
Exploratory behaviors	6.53 (2.08)	0.98 (0.765)	2.08	11.82
Look to person/object	1.96 (1.15)	0.92 (0.003)	0.39	4.57
Look to mother	0.64 (0.65)	0.78 (< 0.001)	0.00	3.64
Play with, examine	3.75 (2.18)	0.97 (0.293)	0.20	9.16
Communicative behaviors	7.19 (3.19)	0.98 (0.919)	0.60	15.26
Vocalization	4.48 (2.85)	0.96 (0.092)	0.21	12.78
Fuss/cry	1.26 (1.73)	0.79 (< 0.001)	0.00	7.61
Smile	1.32 (1.31)	0.88 (< 0.001)	0.00	5.02
Gesture	0.13 (0.30)	0.68 (< 0.001)	0.00	1.48

### 3.3 Maternal Responsiveness

**FIGURE 3:**
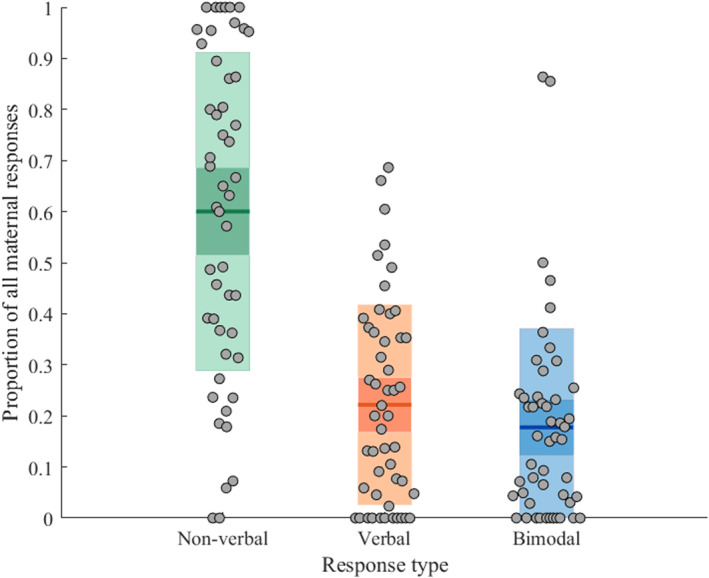
Distribution of maternal responses in each behavioral modality. The middle lines represent the group mean, the inner bands represent the 1.96 standard error of measurement intervals, and the other bands are the ± 1 standard deviation intervals. The gray dots represent individual data points.

Table 4 summarizes overall maternal responsiveness and the proportion of responses that were categorized within each response modality, while Table 5 shows the prevalence of all constituent maternal response behaviors. Figure 3 shows the distribution of responses within each response modality.

**TABLE 4:** Means, standard deviations, medians, quartiles (first, Q1, and third, Q3), and interquartile ranges (IQR) and Shapiro–Wilk test statistics for overall maternal responsiveness and for each response modality. *N* = 50.

	Mean (SD)	*W* (*p* value)	Median	Q1	Q3	IQR
Overall maternal responsiveness	0.62 (0.29)	0.93 (0.006)	0.70	0.36	0.89	0.53
Non‐verbal responsiveness	0.60 (0.31)	0.96 (0.103)	0.64	0.36	0.89	0.53
Verbal responsiveness	0.22 (0.20)	0.93 (0.004)	0.20	0.05	0.36	0.32
Bimodal responsiveness	0.18 (0.19)	0.81 (< 0.001)	0.16	0.03	0.24	0.21

**TABLE 5:** Maternal response behaviors—proportions of all constituent maternal response behaviors. *N* = 50.

Behavior	Mean	SD	Min	Max
Touch (play and affectionate)	0.24	(0.20)	0	1.00
Reposition infant	0.06	(0.07)	0	0.33
Play	0.06	(0.08)	0	0.34
Look to infant	0.06	(0.11)	0	0.60
Smile	0.09	(0.09)	0	0.40
Confirmatory act	0.01	(0.03)	0	0.13
Point	0.04	(0.06)	0	0.25
Social sounds (non‐speech)	0.14	(0.13)	0	0.61
Vocalization	0.30	(0.22)	0	0.73

Mothers responded to an average of 62% (SD = 0.29) of infant behaviors, but there was a wide range between mothers from 6% to 100%. Friedman Test showed significant differences in response modality (*Q* (2) = 23.59, *p* < 0.001). Post‐hoc pairwise Wilcoxon signed rank tests showed that mothers were significantly more likely to engage in non‐verbal behavioral responsiveness than both verbal (*T* = 197.5, *z* = −4.23, *p* < 0.001, *p*_bonferroni_ < 0.001) and bimodal responsiveness (*T* = 147, *z* = −4.74, *p* < 0.001, *p*_bonferroni_ < 0.001), but there was no significant difference between verbal and bimodal responsiveness (*T* = 435, *z* = −1.82, *p* = 0.07, *p*_bonferroni_ = 0.21).

### 3.4 Associations Between Demographic Characteristics and Maternal Responsiveness

Mothers with some formal education were significantly more responsive (median 0.89) than mothers with no formal education (median 0.59; *z* = −2.67, *p* = 0.008, *p*_bonferroni_ = 0.03) and had higher rates of bimodal responsiveness, prior to Bonferonni correction (*z* = −2.01, *p* = 0.044, *p*_bonferroni_ = 0.18). However, the two groups did not differ on non‐verbal (*z* = 1.85, *p* = 0.065, *p*_bonferroni_ = 0.26) or verbal responsiveness (*z* = −1.25, *p* = 0.212, *p*_bonferroni_ = 0.85). Among mothers with formal education, the number of years in school was not associated with overall responsiveness, (*r*_s_ (14) = 0.08, *p* = 0.764). Additionally, maternal age (*r*_s_ (39) = −0.40, *p* = 0.01, *p*_bonferroni_ = 0.06), household size (*r*_s_ (39) = −0.34, *p* = 0.03, *p*_bonferroni_ = 0.17) and mothers' number of children (*r*_s_ (39) = −0.33, *p* = 0.03, *p*_bonferroni_ = 0.21) were all moderately negatively associated with overall maternal responsiveness, although none of these associations survived Bonferroni correction.

### 3.5 Associations Between Infant Physical Growth and Infant Behaviors

Higher infant weight‐for‐length *z*‐score was associated with more looks towards mother (*r*_s_ (48) = 0.41, *p* = 0.004, *p*_bonferroni_ = 0.06). This test was rerun with exclusion of a single outlier (looks to mother > 4 SDs above the mean) and the positive association was maintained, although it did not survive Bonferonni correction (*r*_s_ (47) = 0.39, *p* = 0.006, *p*_bonferroni_ = 0.11). No associations were found with other exploratory behaviors (*r*_s_^2^ (48) ≤ 0.04, *p*s > 0.05). Infants with lower weight‐for‐length *z*‐scores exhibited more communicative behaviors, although this did not survive Bonferroni correction (*r* (48) = −0.32, *p* = 0.023, *p*_bonferroni_ = 0.42). Although no associations were found between the constituent behaviors (vocalizing, fussing, smiling or gesturing) and weight‐for‐length *z*‐scores (*r*_s_^2^ (48) ≤ 0.06, *p*s > 0.05), the association with communicative behaviors did not remain significant when the test was rerun without fussing (*r* (48) = −0.21, *p* = 0.15), suggesting that this behavior was driving the effect. There were no associations between length‐for‐age *z*‐scores and infant behaviors (*r*_s_^2^ (48) ≤ 0.04, *p*s > 0.05).

### 3.6 Associations Between Maternal Responsiveness and Infant Behaviors

Overall levels of maternal responsiveness were not associated with infant number of exploratory (*r*_s_ (48) = −0.09, *p* = 0.51), nor communicative behaviors (*r*_s_ (48) = 0.08, *p* = 0.59). There was a positive association between maternal bimodal responsiveness and infants' communicative behaviors, at a level approaching significance (*r*_s_ (48) = 0.28, *p* = 0.052). As there were two extreme outliers, with values > 3.5 SDs above the mean for bimodal responsiveness (see Figure 3), the correlation between bimodal responsiveness and infant communicative behaviors was rerun with exclusion of these and remained significant but did not survive Bonferroni correction (*r*_s_ (46) = 0.32, *p* = 0.028, *p*_bonferroni_ = 0.22). There were no other significant associations between maternal responsiveness modalities and infant communicative or exploratory behaviors (*r*_s_^2^ < 0.08, *p*s ≥ 0.05).

### 3.7 Power Calculations

Given the preliminary nature of this study and the exploratory analyses conducted, post‐hoc power analyses were performed for significant findings using G*Power v3.1.9.7 (Faul et al. 2007; see Supporting Information S2 for full details). These revealed excellent power (≥ 0.96) for analyses of maternal responsiveness modalities, adequate to moderate power (0.63–0.85) for infant growth‐behavior associations and between maternal bimodal responsiveness and infant communicative behaviors. However, power was inadequate (0.24–0.43) for detecting associations with maternal education, likely due to small subgroup sizes (e.g., *N* = 16 mothers with formal education), suggesting these should be considered preliminary findings.

## 4 Discussion

This study describes the development of a behavioral coding scheme, the Demba Yaal Interaction Scale (DYIS), to assess caregiver responsiveness in a rural area of The Gambia. We adopted a contextually sensitive approach, firstly, by co‐developing the coding scheme across UK researchers familiar with existing coding schemes and Gambian, Mandinka speaking researchers, who consulted on identification of target caregiver and infant behaviors that are appropriate in this culture. Secondly, by maintaining a broad range of target behaviors, particularly in non‐verbal modalities, we sought to reduce the risk of omitting key caregiver behaviors that are less commonly emphasized in research in Minority World settings. We subsequently used the coding scheme to characterize maternal responsiveness behaviors, and to examine the impact of maternal demographic (age, education, household size, number of children) and infant health (physical growth) factors on dyadic interactions.

Notably, the overall rates of maternal responsiveness in our sample were similar to those found in Minority World samples (e.g., USA, Bornstein et al. 2008), which corresponds with cross‐cultural comparisons showing that the degree of responsiveness is similar across Minority and Majority World settings (Broesch et al. 2016). However, mothers in our sample were more likely to use non‐verbal, rather than verbal or bimodal, behaviors. This is also in line with prior research in Majority World contexts, which suggests that caregivers place less emphasis on didactic conversations and infant‐directed talk (Gratier and Devouche 2017; Kuchirko and Tamis‐LeMonda 2019; Richman et al. 1992; Serpell 2017) and that infant‐directed vocalizations are less prevalent in rural, subsistence populations (Cristia 2022). This highlights the importance of examining a broad range of behaviors and modalities when characterizing caregiving in novel contexts, as focusing solely on verbal behaviors would have biased estimates of maternal responsiveness and misclassified mothers in this setting as less responsive.

Although the predominant response modality was non‐verbal (i.e., did not include speech), there was wide variation in the distribution of maternal response types, with some mothers engaging exclusively in non‐verbal behaviors, while others exhibited high rates of verbal responsiveness, with many responses containing speech. This heterogeneity is not unique to this study; prior research has found similar variation in response behaviors even within relatively homogenous samples from the same culture (Bornstein et al. 2008).

### 4.1 Impact of Maternal Demographic Factors

Mothers with a history of formal education were more responsive overall and had higher rates of bimodal responsiveness (prior to Bonferonni correction) than those who had never attended school. Research in other rural, low‐resource communities in Majority World contexts, similarly finds parents who have exposure to formal education tend to adopt a more structured and hierarchical manner, which resembles a classroom dynamic, when interacting with children (Chavajay 2006; Chavajay and Rogoff 2002; Crago et al. 1993; Levine et al. 2012). Perhaps experiences in the classroom shaped mothers' models of adult‐child relationships and promoted greater emphasis on dyadic turn‐taking (Chavajay and Rogoff 2002; Levine et al. 2012) and reciprocal verbal communication (Richman et al. 1992). Number of years in school was not associated with responsiveness, which may be due to the small number of mothers (*N* = 16) who had attended school (post‐hoc power analyses suggested inadequate power to detect an effect with this sample size). It may also be that *any* exposure to the classroom context, regardless of how long, shapes caregivers' views on interacting with children.

It is also possible that mothers with no formal education are particularly vulnerable to poverty related risks, which impacts on their propensity to respond to infant cues. Participation in education is associated with women's autonomy and their ability to contribute to the finances and decision making in their families (Carlson et al. 2015). Recent research from The Gambia suggests that children whose mothers had no formal education were significantly more likely to die or become undernourished by the age of 5 years (Sey‐Sawo et al. 2023; The Gambia Bureau of Statistics 2021). We also highlight in our sample that mothers with no formal education, were, on average, equally likely as mothers with formal education, to respond to infants using unimodal verbal and non‐verbal behaviors. Therefore, understanding the nature of maternal responses in more vulnerable groups may help harness potentially enriching behaviors that they regularly use, rather than promoting behavioral styles that are unnatural for mothers (Morelli et al. 2018; Serpell and Nsamenang 2014).

Moreover, older mothers, those with more children and living in larger households also had lower levels of responsiveness. Prior research suggests that living in overcrowded conditions may decrease maternal responsiveness because they have to stretch limited resources among multiple children and family members (Bornstein et al. 2023; Wachs and Camli 1991). However, these findings may also reflect shared caregiving practices that are common in this setting (Brotherton et al. 2021), where the contribution of other family members puts less pressure on mothers to detect and respond to every infant cue. However, as these preliminary associations did not survive corrections for multiple testing, future work could focus on understanding how the contribution of a wider network of caregivers impacts on maternal responsiveness.

### 4.2 Infant Physical Growth, Maternal Responsiveness, and Infant Behavior

Infants engaged in multiple exploratory and communicative behaviors during the interactions. An association emerged between higher infant weight‐for‐length *z*‐scores and more looks towards mother, although this behavior occurred quite infrequently (see Table 3), limiting variability in the sample and thus interpretation of this association.

There was also an unexpected relationship between *lower* weight‐for‐length *z*‐scores (i.e., poorer physical growth) and more communicative behaviors, which was revealed to be driven by infant fussing. This is in line with other research showing that undernourished infants show more negative emotionality and are harder to soothe (Baker‐Henningham et al. 2009; Lozoff et al. 1998; Wachs et al. 2005). The decision to include fussing/crying as a communicative behavior is in line with prior work (e.g., Alvarenga et al. 2021) and was made on the basis that infant distress signals may be particularly valuable for examining caregiver responses, as they likely elicit intuitive parenting behaviors (Mesman 2010). However, we acknowledge that periods of distress can interfere with other forms of communication. Therefore, it may be necessary to adopt a more granular approach, and separate fussiness/crying from other communicative behaviors, when relating infant communication to other infant factors (e.g., physical growth, health, or later language/cognitive outcomes). Moreover, these findings must be interpreted with some caution as the sub‐sample of the BRIGHT participants studied in this pilot, on average, showed only moderate deviation from healthy physical growth. While nutritional vulnerability may be associated with reduced social and communicative behaviors (Harel‐Gadassi et al. 2020; Sudfeld et al. 2015), meta‐analyses show limited influence of undernutrition on child social competence in non‐severe cases (Suryawan et al. 2022). Therefore, these analyses must be replicated in the full sample, to shed light on how differing severity of child undernutrition may affect their communicative and exploratory behaviors in dyadic interactions and how that, in turn, may shape maternal responses.

On the other hand, infants whose mothers exhibited more bimodal responsiveness showed more communicative behaviors. This finding is akin to research in other low‐income communities in Brazil (Alvarenga et al. 2021) and Ethiopia (Aboud and Alemu 1995), which suggests that mothers' use of bimodal responses were associated with increased child communicative behaviors (including crying and fussing). Moreover, in the Ethiopian sample, heightened verbal responsiveness was associated with infant language scores (Aboud and Alemu 1995). The increased coupling of speech with non‐verbal actions may provide stimulation to increase infant communication, thereby providing a training ground for language acquisition (Alvarenga et al. 2021). These findings are interesting in light of prior findings from BRIGHT, where, using Language Environment Analysis (LENA) technology, we demonstrated that more contingent child‐adult vocal turn taking at 12‐month was associated with more child vocalizations at older ages (Katus et al. 2024). An avenue for future work beyond the current pilot will be to examine relationships between caregiver responsiveness, particularly verbal/bimodal responses, and infant language outcomes.

On the other hand, there was no association between maternal responsiveness and infant exploratory behaviors. This could be a consequence of the recording setup, where infants did not have objects during the 5 min without toys, and mothers actively discouraged them from roaming around the room for the sake of the recording. It may be that, in more naturalistic settings, with more room for exploration, these associations would emerge.

#### 4.2.1 Strengths and Limitations

Our findings suggest that this newly developed coding scheme was a sensitive measure of maternal responsiveness. A particular strength was our co‐creative approach to designing the scheme, and the inclusion of a broad range of caregiver behaviors and response modalities. Speaking to the validity of our coding scheme is the replication of numerous findings from the literature, namely the presence of high rates of non‐verbal behavioral responsiveness and associations between maternal formal education and variations in response behaviors.

However, several important limitations must be considered. Firstly, we were unable to assess joint attention (JA); the toys provided were not suitable for this context and participants did not systematically have an object in place to facilitate JA. This is important as JA is a key interactive characteristic that emerges at this age and is highly relevant for infant learning (Morales et al. 2000). Moreover, the coding scheme only focused on the *frequency* of responses, but did not consider their nature (e.g., the content of maternal speech) or appropriateness. While this made the coding scheme more objective and reduced cultural bias, more research, particularly qualitative work, is needed to help describe what constitutes *sensitive* caregiving in this setting.

Moreover, we were unable to assess the construct validity of our coding scheme against other measures of caregiver responsiveness, largely due to the scarce availability of such measures that would be suitable for this context. An interesting avenue for future research could be to compare our scheme against a coding scheme developed in a Minority World setting to establish whether we would detect higher rates of responsiveness with our contextually‐tailored measure, and if this would have any implications for infant outcomes.

A further limitation is the sole focus on interactions with mothers, in isolation from other caregivers. Our prior work suggests that infants in the BRIGHT cohort have multiple adult caregivers and are frequently in the presence of other children, sibling and non‐sibling (Katus et al. 2024). Therefore, to fully understand the caregiving experience of infants in this context, it may be important to adopt an enlarged family systems perspective (Bornstein 2012). In a similar low‐income setting in Kenya, incorporating interactions with all members of the caregiving environment, compared to assessing maternal responsiveness alone, significantly increased the rate of responsiveness that was measured (Whaley et al. 2002). In addition, older children may be important sources of social input. For instance, for toddlers growing up in non‐industrial Lesotho, language input from other children was shown to be more common than from mothers or other adults (Loukatou et al. 2022). Similarly, future research would benefit from observing interactions in the home, rather than in a standardized laboratory setting, to capture more representative daily dyadic interactions (Tamis–LeMonda et al. 2017). Finally, it is important to re‐iterate that the results presented here are preliminary and based on a small sample size. Future work will involve applying the coding scheme to all participants in the BRIGHT sample.

## 5 Conclusion

In summary, our work highlights the importance of developing assessments that are tailored for specific contexts to study caregiving behaviors. The coding scheme developed in this study was aimed at a specific, rural population in The Gambia and may not necessarily be generalizable to other contexts. However, the methods we describe to develop the coding scheme may serve as a framework for other researchers who aim to create contextually tailored caregiver assessments. Future directions of this work will involve replicating these analyses in the full BRIGHT cohort and examining the impact of maternal responsiveness on infant language, cognitive and neural development.

## Author Contributions

**Helena‐Céline Stevelt:** conceptualization (lead), writing – original draft preparation (lead), formal analysis (lead), writing – review and editing, methodology, data curation, visualization. **Ebrima Mbye:** methodology, project administration, investigation, writing – review and editing. **Ebou Touray:** methodology, project administration, investigation, writing – review and editing. **Tijan Fadera:** methodology, investigation, writing – review and editing. **Mariama Saidykhan:** methodology, investigation, writing – review and editing. **Ousman Kambi:** methodology, investigation, writing review and editing. **Eirini Papageorgopoulou:** methodology, validation, writing – review editing. **Samantha McCann:** project administration, investigation, writing – review and editing. **Clare E. Elwell:** funding acquisition, project administration, supervision, writing – review and editing. **Sophie E. Moore:** funding acquisition, project administration, supervision, writing – review editing. **Sarah Lloyd‐Fox:** conceptualization, funding acquisition, supervision, methodology, project administration, writing – review and editing. **Bosiljka Milosavljevic:** conceptualization, supervision, methodology, data curation, writing – original draft preparation (support), writing – review and editing.

## Conflicts of Interest

The authors declare no conflicts of interest.

## Supporting information


Supporting Information S1

Supporting Information S2

## Data Availability

Data supporting this paper will be made available subject to established data sharing agreements.
