# Supplementary material for: A Contextual Approach to Characterizing Caregiver Responsiveness in a Rural Area of The Gambia
Source: Infancy. 2025 Oct 1;30(5):e70047. doi: 10.1111/infa.70047 (PMC12487999; doi:10.1111/infa.70047)
Supplement: Supplementary file 1 — Supporting Information S1 [file INFA-30-0-s001.docx]

STROBE Statement—checklist of items that should be included in reports of observational studies

|  | Item No. | Recommendation | Page  No. | Relevant text from manuscript |
| --- | --- | --- | --- | --- |
| **Title and abstract** | 1 | (*a*) Indicate the study’s design with a commonly used term in the title or the abstract | 2 | This study describes the development of the “Demba Yaal Interaction Scale (DYIS)”, a behavioural micro-coding scheme to assesses caregiver responsiveness in a rural, low-resource, collectivist caregiving community in The Gambia.  […]  The scheme was piloted on 5-minute videorecorded mother-infant interactions, when infants were aged 12-months (*N*=50, 48% female). |
|  |  | (*b*) Provide in the abstract an informative and balanced summary of what was done and what was found | 2 | We adopted a contextually sensitive approach by co-creating the scheme partnering Gambian researchers, familiar with the caregiving context, and UK researchers familiar with behavioural coding. The scheme was piloted on 5-minute videorecorded mother-infant interactions, when infants were aged 12-months (*N*=50, 48% female). There were substantial individual differences in maternal responsiveness levels. Modality-wise, responses were most likely to be non-verbal, compared to verbal or bimodal. Mothers with some formal education were significantly more responsive and more readily engaged in bimodal responsiveness. Negative associations between these interactive behaviours and maternal demographic and socioeconomic variables (age, number of children, household size) were present but did not remain significant after correction for multiple comparisons. |
| Introduction | | | |  |
| Background/rationale | 2 | Explain the scientific background and rationale for the investigation being reported | 3-7 | *Note: there is too much text to summarise as the background and rationale are distributed throughout the introduction, which spans 4 pages.* |
| Objectives | 3 | State specific objectives, including any prespecified hypotheses | 8 | The aim of the present study is to describe the development of this behavioural coding scheme of caregiver responsiveness. Furthermore, we use a subset of the BRIGHT participants to pilot this coding scheme to characterise maternal and infant behaviours and to examine whether contextually specific demographic and SES (lower educational attainment, larger number of children, larger household size) and poorer infant health (reduced physical growth) factors negatively impact on mother-infant dyadic behaviours. |
| Methods | | | |  |
| Study design | 4 | Present key elements of study design early in the paper | 8 | The battery of assessments in the BRIGHT project involved collecting data on maternal-infant interactions during recorded, free play sessions administered at, 1-, 5-, 8-, 12-, 18- and 24-months of age (Lloyd-Fox et al., 2024). In the present study, we use data collected at the 12-month time point to develop a contextually sensitive behavioural coding scheme of caregiver responsiveness for this setting. We named the scheme the “Demba Yaal Interaction Scale (DYIS)”, using the Mandinka word for family (demba yaal).  […]  Furthermore, we use a subset of the BRIGHT participants to pilot this coding scheme to characterise maternal and infant behaviours and to examine whether contextually specific demographic and SES (lower educational attainment, larger number of children, larger household size) and poorer infant health (reduced physical growth) factors negatively impact on mother-infant dyadic behaviours. |
| Setting | 5 | Describe the setting, locations, and relevant dates, including periods of recruitment, exposure, follow-up, and data collection | 8-9 | Families were recruited during an antenatal clinic visit at the Medical Research Council Unit The Gambia at the London School of Hygiene and Tropical Medicine (MRCG at LSHTM; <https://www.lshtm.ac.uk/research/units/mrc-gambia>) field station in Keneba, West Kiang. To avoid confounds with linguistic translation of assessments, only families of the Mandinka ethnic group, the majority in the region (Hennig et al., 2017) were recruited. The BRIGHT sample consisted of 222 families; infants were included if they were born at 37-42 weeks’ gestation and not diagnosed with any neurological difficulties postnatally. For full detail of BRIGHT recruitment and study visits please see Lloyd-Fox et al. (2024). To pilot the coding scheme, a subset of 50 mother-infant dyads (48% female) was randomly selected from the full sample that attended the 12-month visit (*N* = 188). |
| Participants | 6 | 1. *Cohort study*—Give the eligibility criteria, and the sources and methods of selection of participants. Describe methods of follow-up   *Case-control study*—Give the eligibility criteria, and the sources and methods of case ascertainment and control selection. Give the rationale for the choice of cases and controls  *Cross-sectional study*—Give the eligibility criteria, and the sources and methods of selection of participants | 9 | The BRIGHT sample consisted of 222 families; infants were included if they were born at 37-42 weeks’ gestation and not diagnosed with any neurological difficulties postnatally. |
|  |  | (*b*) *Cohort study*—For matched studies, give matching criteria and number of exposed and unexposed  *Case-control study*—For matched studies, give matching criteria and the number of controls per case |  |  |
| Variables | 7 | Clearly define all outcomes, exposures, predictors, potential confounders, and effect modifiers. Give diagnostic criteria, if applicable | 16-17 | ***Maternal demographic characteristics***  Maternal demographic variables were collected using questionnaires at the 7-14-day visit. Factors included in the current study were mothers’ age, number of children, household size (by asking how many adults and children lived in their household), and formal education. Maternal education was examined both as number of years in school and, because many mothers did not have any formal schooling, a dichotomous variable indicating whether the mother had any formal education (yes/no).  ***Infant anthropometric measures***  Infant weight and length were collected at the 12-month visit. Length was measured with a Harpenden Infantometer length board (Holtain Ltd) with a precision of 0.1cm and weight was measured using an electronic baby scale (Model 336, SECA) with a precision of 0.01kg. Measures were taken three times and the mean of those were used in analyses. Infants’ length-for-age and weight-for-length sex-adjusted z-scores were computed according to WHO reference norms (WHO Multicentre Growth Reference Study Group, 2006). These scores can be used to identify infants that are stunted (length-for-age z-score < -2 SD) or wasted (weight-for-length z-score < -2 SD). |
| Data sources/ measurement | 8* | For each variable of interest, give sources of data and details of methods of assessment (measurement). Describe comparability of assessment methods if there is more than one group | 16-17 | *See text in item 7* |
| Bias | 9 | Describe any efforts to address potential sources of bias | N/A | *Note: participants were selected at random from a larger sample. However, this may not eliminate all bias in the larger recruitment protocol.* |
| Study size | 10 | Explain how the study size was arrived at | 9 | This sample size was based on published studies using similar coding of maternal responsiveness, which ranged from 20-40 participants (Bornstein et al., 1999; Kärtner et al., 2008; Masur et al., 2005; Richman et al., 1992; Tamis-LeMonda et al., 2001). |

Continued on next page

| Quantitative variables | 11 | Explain how quantitative variables were handled in the analyses. If applicable, describe which groupings were chosen and why | 11-17 | *Note: there is too much text to summarise here, as the description of quantitative variables is distributed in different parts throughout the methods section, which spans 6 pages.* |
| --- | --- | --- | --- | --- |
| Statistical methods | 12 | (*a*) Describe all statistical methods, including those used to control for confounding | 17-18 | ***Data analysis strategy***  The first objective in data analysis was to comprehensively characterise maternal and infant behaviours. For infants, we examined overall activity levels, rates of communicative and exploratory behaviours, and rates of each constituent behaviour. For maternal behaviours, we examined overall responsiveness, rates of verbal, non-verbal, and bimodal responsiveness, and all constituent behaviours. Friedman’s test was used to compare the rates of responses in each modality. As a significant difference emerged (see results) pairwise Wilcoxon signed rank tests were used to identify the modalities that significantly differed from each other.  To examine the contribution of physical growth to infant behaviour, correlations were run between infant weight-for-length and length-for-age z-scores, the number of exploratory and communicative behaviours. As these analyses are exploratory, we also ran correlations with each constituent behaviour. To control for potential effects of infant distress on the relationship between physical health and communication, a supplementary analysis was run excluding fussing from the communicative behaviour category. All variables were checked for normality, and if both variables fit assumptions of normality, Pearson’s correlation was run, otherwise non-parametric Spearman’s rho correlations were used.  To assess the impact of maternal education on responsiveness, Mann-Whitney U tests were run to compare overall responsiveness and each response modality between mothers who had some vs. no formal education. For mothers who had some formal education, Spearman Rho correlation was used to examine the association between the number of years of schooling and overall responsiveness. Similarly, Spearman Rho correlation was used to examine the association between maternal age, number of children, household size and overall maternal responsiveness. Finally, Spearman Rho correlations were used to assess associations between overall maternal responsiveness, response modalities and infant number of exploratory and communicative behaviours. Given the exploratory nature of these analyses, significance is reported using both uncorrected and Bonferonni-adjusted *p*-values. |
|  |  | (*b*) Describe any methods used to examine subgroups and interactions | N/A | N/A |
|  |  | (*c*) Explain how missing data were addressed | N/A | *Note: As this was a pilot study, only participants who had available data were selected and we did not have to handle missing data.* |
|  |  | (*d*) *Cohort study*—If applicable, explain how loss to follow-up was addressed  *Case-control study*—If applicable, explain how matching of cases and controls was addressed  *Cross-sectional study*—If applicable, describe analytical methods taking account of sampling strategy | N/A | N/A |
|  |  | (*e*) Describe any sensitivity analyses | N/A | *Note: Sensitivity analyses were not conducted, and this has been addressed in the discussion:*  *(pg. 31)*  Moreover, we were unable to assess the construct validity of our coding scheme against other measures of caregiver responsiveness, largely due to the scarce availability of such measures that would be suitable for this context. An interesting avenue for future research could also be to compare our scheme against a coding scheme developed in a Minority World setting to establish whether we would detect higher rates of responsiveness with our contextually-tailored measure, and if this would have any implications for infant outcomes. |
| Results | | | | |
| Participants | 13* | (a) Report numbers of individuals at each stage of study—eg numbers potentially eligible, examined for eligibility, confirmed eligible, included in the study, completing follow-up, and analysed | N/A | *Note: As this was a pilot study, only a subset of N=50 participants were selected at random from a larger group of N=222 participants.* |
|  |  | (b) Give reasons for non-participation at each stage | N/A | See point 13 a |
|  |  | © Consider use of a flow diagram | N/A | See point 13 a |
| Descriptive data | 14* | (a) Give characteristics of study participants (eg demographic, clinical, social) and information on exposures and potential confounders | 19 | *These details are summarised in Table 2* |
|  |  | (b) Indicate number of participants with missing data for each variable of interest | N/A | *Note: as this was a pilot study where only participants with available data were selected, we did not have to handle missing data.* |
|  |  | (c) *Cohort study*—Summarise follow-up time (eg, average and total amount) |  |  |
| Outcome data | 15* | *Cohort study*—Report numbers of outcome events or summary measures over time |  |  |
|  |  | *Case-control study—*Report numbers in each exposure category, or summary measures of exposure |  |  |
|  |  | *Cross-sectional study—*Report numbers of outcome events or summary measures | 20-22 | *These details are summarised in Tables 3 and 4.* |
| Main results | 16 | (*a*) Give unadjusted estimates and, if applicable, confounder-adjusted estimates and their precision (eg, 95% confidence interval). Make clear which confounders were adjusted for and why they were included | 23-25 | Mothers responded to an average of 62% *(SD*=0.29) of infant behaviours, but there was a wide range between mothers from 6-100%. Friedman Test showed significant differences in response modality (*Q(2)* = 23.59, *p* < .001). Post-hoc pairwise Wilcoxon signed rank tests showed that mothers were significantly more likely to engage in non-verbal behavioural responsiveness than both verbal (*T* = 197.5 =, *z* = -4.23, *p* < .001, *p*_bonferroni_ < .001) and bimodal responsiveness (*T* = 147, *z* = -4.74, *p* < .001, *p*_bonferroni_ < .001), but there was no significant difference between verbal and bimodal responsiveness (*T* = 435, *z* = -1.82, *p* = .07, *p*_bonferroni_ = .21). *Associations between demographic characteristics and maternal responsiveness* Mothers with some formal education were significantly more responsive (median .89) than mothers with no formal education (median .59) (*z* = -2.67, *p* = .008, *p*_bonferroni_ = .03) and had higher rates of bimodal responsiveness, prior to Bonferonni correction (*z* = -2.01, *p* = .044, *p*_bonferroni_ = .18). However, the two groups did not differ on non-verbal (*z* = 1.85, *p* = .065, *p*_bonferroni_ = .26) or verbal responsiveness (*z* = -1.25, *p* = .212, *p*_bonferroni_ = .85). Among mothers with formal education, the number of years in school was not associated with overall responsiveness, (*r*_s_(14) = .08, *p* = .764). Additionally, maternal age (*r*_s_(39) = -.40, *p* = .01, *p*_bonferroni_ = .06), household size (*r*_s_(39) = -.34, *p* = .03, *p*_bonferroni_ = .17) and mothers’ number of children (*r*_s_(39) = -.33, *p* = .03, *p*_bonferroni_ = .21) were all moderately negatively associated with overall maternal responsiveness, although none of these associations survived Bonferroni correction. *Associations between infant physical growth and infant behaviours* Higher infant weight-for-length z-score was associated with more looks towards mother (*r_s_*(48) = .41, *p* = .004, *p_bonferroni_* = .06). This test was rerun with exclusion of a single outlier (looks to mother > 4 SDs above the mean) and the positive association was maintained, although it did not survive Bonferonni correction (*r*_s_(47) = .39, *p* = .006, *p_bonferroni_* = .11). No associations were found with other exploratory behaviours (*r*_s_^2^ (48) ≤ .04, *p*s > .05). Infants with lower weight-for-length z-scores exhibited more communicative behaviours, although this did not survive Bonferroni correction (*r*(48) = -0.32, *p* = .023, *p_bonferroni_* = .42). Although no associations were found between the constituent behaviours (vocalising, fussing, smiling or gesturing) and weight-for-length z-scores (*r*_s_^2^(48) ≤ .06, *p*s > .05), the association with communicative behaviours did not remain significant when the test was rerun without fussing (*r*(48) = -0.21, *p* = .15), suggesting that this behaviour was driving the effect. There were no associations between length-for-age z-scores and infant behaviours (*r*_s_^2^(48) ≤ .04, *p*s > .05). *Associations between maternal responsiveness and infant behaviours* Overall levels of maternal responsiveness were not associated with infant number of exploratory (*r*_s_(48) = -0.09, *p* = .51), nor communicative behaviours (*r*_s_(48) = 0.08, *p* = .59). There was a positive association between maternal bimodal responsiveness and infants’ communicative behaviours, at a level approaching significance (*r*_s_(48) = .28, *p* = .052). As there were two extreme outliers, with values >3.5 SDs above the mean for bimodal responsiveness (see Figure 3), the correlation between bimodal responsiveness and infant communicative behaviours was rerun with exclusion of these and remained significant but did not survive Bonferroni correction (*r*_s_(46) = .32, *p* = .028, *p_bonferroni_* = .22). There were no other significant associations between maternal responsiveness modalities and infant communicative or exploratory behaviours (*r*_s_^2^ < .08, *p*s ≥ .05). |
|  |  | (*b*) Report category boundaries when continuous variables were categorized | 19-22 | Tables 2, 3 and 4. |
|  |  | © If relevant, consider translating estimates of relative risk into absolute risk for a meaningful time period | N/A |  |

Continued on next page

| Other analyses | 17 | Report other analyses done—eg analyses of subgroups and interactions, and sensitivity analyses | N/A |  |
| --- | --- | --- | --- | --- |
| Discussion | | | | |
| Key results | 18 | Summarise key results with reference to study objectives | 25-26 | This study describes the development of a behavioural coding scheme, the Demba Yaal Interaction Scale (DYIS), to assess caregiver responsiveness in a rural area of The Gambia. We adopted a contextually sensitive approach, firstly, by co-developing the coding scheme across UK researchers familiar with existing coding schemes and Gambian, Mandinka speaking researchers, who consulted on identification of target caregiver and infant behaviours that are appropriate in this culture. Secondly, by maintaining a broad range of target behaviours, particularly in non-verbal modalities, we sought to reduce the risk of omitting key caregiver behaviours that are less commonly emphasised in research in Minority World settings. We subsequently used the coding scheme to characterise maternal responsiveness behaviours, and to examine the impact of maternal demographic (age, education, household size, number of children) and infant health (physical growth) factors on dyadic interactions.  Notably, the overall rates of maternal responsiveness in our sample were similar to those found in Minority World samples (e.g., USA, Bornstein et al., 2008), which corresponds with cross-cultural comparisons showing that the degree of responsiveness is similar across Minority and Majority World settings (Broesch et al., 2016). However, mothers in our sample were more likely to use non-verbal, rather than verbal or bimodal, behaviours. This is also in line with prior research in Majority World contexts, which suggests that caregivers place less emphasis on didactic conversations and infant-directed talk (Gratier & Devouche, 2017; Kuchirko & Tamis-LeMonda, 2019; Richman et al., 1992; Serpell, 2017) and that infant-directed vocalisations are less prevalent in rural, subsistence populations (Cristia, 2022). This highlights the importance of examining a broad range of behaviours and modalities when characterising caregiving in novel contexts, as focusing solely on verbal behaviours would have biased estimates of maternal responsiveness and misclassified mothers in this setting as less responsive.  Although the predominant response modality was non-verbal (i.e. did not include speech), there was wide variation in the distribution of maternal response types, with some mothers engaging exclusively in non-verbal behaviours, while others exhibited high rates of verbal responsiveness, with many responses containing speech. This heterogeneity is not unique to this study; prior research has found similar variation in response behaviours even within relatively homogenous samples from the same culture (Bornstein et al., 2008). |
| Limitations | 19 | Discuss limitations of the study, taking into account sources of potential bias or imprecision. Discuss both direction and magnitude of any potential bias | 30-31 | However, several important limitations must be considered. Firstly, we were unable to assess joint attention (JA); the toys provided were not suitable for this context and participants did not systematically have an object in place to facilitate JA. This is important as JA is a key interactive characteristic that emerges at this age and is highly relevant for infant learning (Morales et al., 2000). Moreover, the coding scheme only focused on the *frequency* of responses, but did not consider their nature (e.g., the content of maternal speech) or appropriateness. While this made the coding scheme more objective and reduced cultural bias, more research, particularly qualitative work, is needed to help describe what constitutes *sensitive* caregiving in this setting.  Moreover, we were unable to assess the construct validity of our coding scheme against other measures of caregiver responsiveness, largely due to the scarce availability of such measures that would be suitable for this context. An interesting avenue for future research could also be to compare our scheme against a coding scheme developed in a Minority World setting to establish whether we would detect higher rates of responsiveness with our contextually-tailored measure, and if this would have any implications for infant outcomes.  A further limitation is the sole focus on interactions with mothers, in isolation from other caregivers. Our prior work suggests that infants in the BRIGHT cohort have multiple adult caregivers and are frequently in the presence of other children, sibling and non-sibling (Katus et al., 2024). Therefore, to fully understand the caregiving experience of infants in this context, it may be important to adopt an enlarged family systems perspective (Bornstein, 2012). In a similar low-income setting in Kenya, incorporating interactions with all members of the caregiving environment, compared to assessing maternal responsiveness alone, significantly increased the rate of responsiveness that was measured (Whaley et al., 2002). In addition, older children may be important sources of social input. For instance, for toddlers growing up in non-industrial Lesotho, language input from other children was shown to be more common than from mothers or other adults (Loukatou et al., 2022). Similarly, future research would benefit from observing interactions in the home, rather than in a standardised laboratory setting, to capture more representative daily dyadic interactions (Tamis‐LeMonda et al., 2017). Finally, it is important to re-iterate that the results presented here are preliminary and based on a small sample size. Future work will involve applying the coding scheme to all participants in the BRIGHT sample. |
| Interpretation | 20 | Give a cautious overall interpretation of results considering objectives, limitations, multiplicity of analyses, results from similar studies, and other relevant evidence | 26-30 | *Note: there is too much content to summarise in this table, as this spans the discussion over 4 pages.* |
| Generalisability | 21 | Discuss the generalisability (external validity) of the study results | 31-32 | In summary, our work highlights the importance of developing assessments that are tailored for specific contexts to study caregiving behaviours. The coding scheme developed in this study was aimed at a specific, rural population in The Gambia and may not necessarily be generalisable to other contexts. However, the methods we describe to develop the coding scheme may serve as a framework for other researchers who aim to create contextually tailored caregiver assessments. |
| Other information | |  | | |
| Funding | 22 | Give the source of funding and the role of the funders for the present study and, if applicable, for the original study on which the present article is based | 1 | This project is funded by the Bill and Melinda Gates  Foundation (grants OPP1061089 and OPP1127625). The Nutrition Theme at MRCG is  funded by the MRC & the Department for International Development (DFID) under the  MRC/DFID Concordat agreement (MRC Programme MC-A760-5QX00). SEM is funded by  a Wellcome Trust Senior Research Fellowship (220225/Z/20/Z). SLF is funded by a UKRI  Future Leaders Fellowship (grant number MR/S018425/1)*.* BM was supported by an ESRC  Secondary Data Analysis Initiative Grant (ES/V016601/1). |

*Give information separately for cases and controls in case-control studies and, if applicable, for exposed and unexposed groups in cohort and cross-sectional studies.

**Note:** An Explanation and Elaboration article discusses each checklist item and gives methodological background and published examples of transparent reporting. The STROBE checklist is best used in conjunction with this article (freely available on the Web sites of PLoS Medicine at http://www.plosmedicine.org/, Annals of Internal Medicine at http://www.annals.org/, and Epidemiology at http://www.epidem.com/). Information on the STROBE Initiative is available at www.strobe-statement.org.
