# Supplementary material for: A Contextual Approach to Characterizing Caregiver Responsiveness in a Rural Area of The Gambia
Source: Infancy. 2025 Oct 1;30(5):e70047. doi: 10.1111/infa.70047 (PMC12487999; doi:10.1111/infa.70047)
Supplement: Supplementary file 2 — Supporting Information S2 [file INFA-30-0-s002.docx]

**Supplementary Material: Power calculations**

Post-hoc power analyses were conducted using G*Power version 3.1.9.7 (Faul et al., 2007)

***Maternal responsiveness***
G*Power *Test family*: F tests *Statistical test*: ANOVA: Repeated measures, within factors

For the comparison of maternal responsiveness modalities using Friedman’s test (N = 50), the observed effect size was 0.49 (f = √(Q/(N×(k-1))) = √(23.59/(50×(3-1))) = .486). At α = 0.05, the achieved power was 1.00, indicating excellent power to detect this effect.

*Note: Power was estimated using G*Power's repeated measures ANOVA approximation, as direct calculations for Friedman's test are not available.*

For the pairwise comparisons between responsiveness modalities using Wilcoxon signed-rank tests, effect sizes were calculated as *d* = z/√N, analyses revealed the following power values: non-verbal vs verbal (*d* = 0.60, at α = 0.05 power = 0.99; at Bonferroni-corrected α = 0.017, power = 0.96), non-verbal vs bimodal (*d = 0.67, at α = 0.05 power = 1.00; at Bonferroni-corrected α = 0.017, power = 0.99*), and verbal vs bimodal (*d* = 0.26, at α = 0.05 power = 0.43; at Bonferroni-corrected α = 0.017, power = 0.26). This indicates excellent power for the first two analyses, but inadequate power for detecting differences between verbal and bimodal responsiveness.

*Note: Power approximated using paired t-test models, as direct calculations for Wilcoxon signed-rank tests are not available.*

### ***Associations between demographic characteristics and maternal responsiveness***

G*Power *Test family*: t tests *Statistical test*: Means: Difference between two independent means (two groups)

For overall responsiveness comparing mothers with vs. without formal education (*N* = 41, *n* = 25 without education, *n* = 16 with education), the observed effect size was *d* = 0.42 (calculated as z/√N = 2.67/√41). At α = 0.05, the achieved power was 0.36, indicating low power to detect this effect.

For bimodal responsiveness differences by education, the observed effect size was *d* = 0.31 (z = 2.01, *d* = 2.01/√41). At α = 0.05, the achieved power was 0.24; at Bonferroni-corrected α = 0.18, the achieved power was 0.24, indicating low power to detect this effect.

***Associations between infant physical growth and infant behaviours***

G*Power Test family: Exact Statistical test: Correlation: Bivariate normal model

Power calculations for associations between infant physical growth and infant behaviours used Bonferroni-corrected α = 0.003 to account for multiple comparisons.

For WLZ and looks to mother (*N* = 50), the observed effect size was *r* = 0.39-0.41. At α = 0.003, the achieved power was 0.81-0.85 (0.43-0.50 with Bonferroni correction), indicating adequate power to detect this effect.

For WLZ and communicative behaviours (*N* = 50), the observed effect size was *r* = -0.32. At α = 0.003, the achieved power was 0.63 (0.23 with Bonferroni correction), indicating moderate power to detect this effect.

*Note: Power calculations used bivariate normal model as approximation, since Spearman's rho model is not available in G*Power.*

***Associations between maternal responsiveness and infant behaviours***

G*Power *Test family*: Exact *Statistical test*: Correlation: Bivariate normal model

For associations between maternal bimodal responsiveness and infant communicative behaviours (*N* = 50), the observed effect size was *r_s_* = 0.32. At α = 0.05, the achieved power was 0.63 (0.36 with Bonferroni correction at α = 0.0083), indicating moderate power to detect this effect.

*Note: Power calculations used bivariate normal model as approximation, as Spearman’s rho model is not available.*
